# *Mycobacterium tuberculosis* resistance to antituberculosis drugs in Mozambique*, **

**DOI:** 10.1590/S1806-37132014000200007

**Published:** 2014

**Authors:** Germano Manuel Pires, Elena Folgosa, Ndlovu Nquobile, Sheba Gitta, Nureisha Cadir

**Affiliations:** National Institute of Health, Ministry of Health, Maputo, Mozambique; Eduardo Mondlane University School of Medicine, Maputo, Mozambique; African Field Epidemiology Network, Kampala, Uganda; African Field Epidemiology Network, Kampala, Uganda; National Tuberculosis Referral Laboratory, Maputo, Mozambique

**Keywords:** Extensively drug-resistant tuberculosis, Tuberculosis, Tuberculosis, multidrug-resistant

## Abstract

**OBJECTIVE:**

To determine the drug resistance profile of *Mycobacterium tuberculosis* in Mozambique.

**METHODS:**

We analyzed secondary data from the National Tuberculosis Referral Laboratory, in the city of Maputo, Mozambique, and from the Beira Regional Tuberculosis Referral Laboratory, in the city of Beira, Mozambique. The data were based on culture-positive samples submitted to first-line drug susceptibility testing (DST) between January and December of 2011. We attempted to determine whether the frequency of DST positivity was associated with patient type or provenance.

**RESULTS:**

During the study period, 641 strains were isolated in culture and submitted to DST. We found that 374 (58.3%) were resistant to at least one antituberculosis drug and 280 (43.7%) were resistant to multiple antituberculosis drugs. Of the 280 multidrug-resistant tuberculosis cases, 184 (65.7%) were in previously treated patients, most of whom were from southern Mozambique. Two (0.71%) of the cases of multidrug-resistant tuberculosis were confirmed to be cases of extensively drug-resistant tuberculosis. Multidrug-resistant tuberculosis was most common in males, particularly those in the 21-40 year age bracket.

**CONCLUSIONS:**

*M*. *tuberculosis* resistance to antituberculosis drugs is high in Mozambique, especially in previously treated patients. The frequency of *M*. *tuberculosis* strains that were resistant to isoniazid, rifampin, and streptomycin in combination was found to be high, particularly in samples from previously treated patients.

## Introduction

Tuberculosis (TB) remains a serious public health problem in many low- and middle-income countries in Africa, Asia, and the former Soviet Union.^(^ ^1^ ^)^ According to the World Health Organization, nearly 9 million new TB cases are recorded globally each year, 4 million of which are infectious TB cases. In many countries, the number of TB cases has quadrupled, despite the implementation of effective strategies to combat the disease.^(^ ^2^ ^)^

Not only has the number of studies of antituberculosis drug resistance increased (from 1 in 2008 to 10 in 2011), but the number of countries providing representative drug resistance data has also increased (from 19 to 22), Mozambique being one such country.^(^ ^3^ ^)^ Treatment failure, poor adherence to treatment, and spontaneous mutations in *Mycobacterium tuberculosis* strains have contributed to the emergence of new multidrug-resistant TB (MDR-TB) cases, which can later develop into extensively drug-resistant TB (XDR-TB) cases.^(^ ^4^ ^-^ ^6^ ^)^ Recent drug resistance studies have identified high rates of MDR-TB in southern Africa, and 69 countries (including Mozambique) had reported at least one case of XDR-TB by the end of 2010.^(^ ^2^ ^,^ ^7^ ^,^ ^8^ ^)^ In Mozambique, drug-resistant TB is thought to be a major problem. The 2008 Mozambican national antituberculosis drug resistance survey showed that, of all MDR-TB cases, 3.5% were newly diagnosed TB cases and 11.2% occurred among individuals who had previously been treated for TB; in 2011, 47,452 cases of all forms of TB were detected,^(^ ^3^ ^)^ although none were reported to be cases of XDR-TB.

Because of the increasing number of cases of MDR-TB^(^ ^4^ ^)^ and the emergence of XDR-TB in Mozambique, we sought to evaluate M. tuberculosis resistance to antituberculosis drugs in previously treated and untreated TB patients in Mozambique. We also sought to determine the magnitude of antituberculosis drug resistance in the country, in order to inform the National Tuberculosis Control Program of the efficacy of TB control measures and treatment, as well as to design effective treatment regimens and strategies for all TB patients in the country.

## Methods

This was a cross-sectional study based on secondary laboratory data for the period of January to December of 2011. The data were based on 641 positive TB cultures from the National Tuberculosis Referral Laboratory, located in the city of Maputo, Mozambique, and the Beira Regional Tuberculosis Referral Laboratory, located in the city of Beira, Mozambique. These two laboratories serve all 11 of the provinces of Mozambique.

An MDR-TB case was defined as an individual infected with an isolate resistant to at least isoniazid and rifampin, confirmed cases of XDR-TB being excluded.^(^ ^9^ ^-^ ^11^ ^)^ Cases of MDR-TB resistant to a fluoroquinolone and a second-line injectable drug other than streptomycin were defined as XDR-TB cases.^(^ ^12^ ^-^ ^15^ ^)^ If an MDR-TB or XDR-TB patient was registered for treatment and had never received TB treatment for longer than 4 weeks, the patient was considered to have primary drug resistance. Patients with MDR-TB/XDR-TB undergoing retreatment and having had the first episode of TB before 2011 were assumed to have acquired drug resistance.

All samples underwent smear microscopy and culture. All culture-positive samples underwent first-line drug susceptibility testing (DST), which was performed by means of the ratio method. Of the MDR-TB samples, 71 were sent to the Supranational TB Referral Laboratory in Milan, Italy, for second-line DST.

Secondary data from the National Tuberculosis Referral Laboratory (demographic data and DST results) were collected from a WixDisa database (version 04.16.04.652; Disa, South Africa) and the laboratory record book, whereas those from the Beira Regional Tuberculosis Referral Laboratory were retrieved from a Microsoft Excel database. All positive culture results and DST results were entered into an Epi Info 3.5.1 database and analyzed. We calculated ORs and their 95% CIs in order to determine the association of previously treated and untreated patients with the results of DST.

The study was reviewed and approved by the Research Ethics Committee of the National Institute of Health, in Maputo, Mozambique. Informed consent was not required, because the study was based on secondary data and we had no access to any identifying patient information.

## Results

A total of 641 TB culture-positive samples (561 samples from the National Tuberculosis Referral Laboratory and 80 from the Beira Regional Tuberculosis Referral Laboratory) were analyzed during the study period. Table 1 shows the distribution of MDR-TB and XDR-TB patients by gender and age bracket.

**Table 1:** Distribution of multidrug-resistant tuberculosis and extensively drug-resistant tuberculosis patients, by gender and age bracket.

Age bracket (years)	MDR-TB patients			XDR-TB patients		
	(n = 280)			(n = 2)		
	Gender		Total	Gender		Total
	Female	Male		Female	Male	
	(n)	(n)		(n)	(n)	
0-20	16	3	19			
21-40	92	99	191	1	1	2
41-60	20	36	56			
> 60	1	4	5			
Missing data	3	6	9			
Total	132	148	280	1	1	2

**MDR-TB:** : multidrug-resistant tuberculosis

**XDR-TB:** : extensively drug-resistant tuberculosis

Of the 641 samples, 430 (67.1%) were from previously treated TB patients and 280 (43.7%) were from MDR-TB patients (Table 2). Of those 280 samples, 148 (53%) were from males and 191 (68.2%) were from individuals in the 21-40 year age bracket. There were 2 XDR-TB patients, both in the 21-40 year age bracket.

**Table 2:** Distribution of multidrug-resistant tuberculosis and non-multidrug-resistant tuberculosis patients, by history of tuberculosis treatment.

Treatment history	MDR-TB patients	Non-MDR-TB patients	OR	p
	n (%)	n (%)		
Previous treatment	184 (65.7)	172 (47.6)	2.06 (1.47-2.88)	< 0.001
No previous treatment	96 (34.3)	185 (51.2)		
Missing data		4 (1.1)		
Total	280 (100)	361 (100)		

**MDR-TB:** : multidrug-resistant tuberculosis

**Figure 1:**
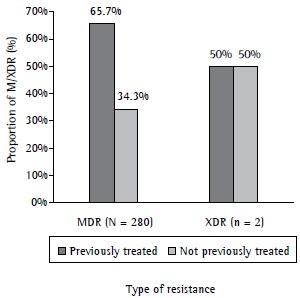
Proportion of multidrug-resistant tuberculosis (MDR) and extensively drug-resistant tuberculosis (XDR) cases by patient treatment history.

Of the MDR-TB samples that were sent to the Supranational TB Referral Laboratory for second-line DST, 2 were confirmed to be XDR-TB samples; of those, 1 was from a previously treated patient, and 1 was from a previously untreated patient (Figure 1).

Among of the 641 samples evaluated, the most common drug resistance pattern was monoresistance to isoniazid (in 5.8%), followed by monoresistance to rifampin (in 3.4%). Table 3 shows the distribution of specific drug resistance patterns by history of TB treatment.

**Table 3:** Distribution of specific drug resistance patterns, by history of tuberculosis treatment.

Monoresistance			Treatment history				OR (95% CI)	p
			No previous treatment		Previous treatment			
			(n = 211)		(n = 430)			
Drug	n	%	n	%	n	%		
H	37	56.9	17	53.1	20	60.6	1.36 (0.45-4.09)	0.720
R	22	33.8	10	31.3	12	36.4	1.26 (0.40-4.00)	0.862
S	2	3.1	1	3.1	1	3.0	0.97 (0.03-37.42)	1.000
E	4	6.2	4	12.5	0	0.0	-	0.053
Total	65	100.0	32	100.0	33	100.0		

H : isoniazid

R : rifampin

S : streptomycin

E : ethambutol

Table 4 shows the distribution of multidrug resistance patterns by history of TB treatment. Most of the *M*. *tuberculosis* samples, particularly those from previously treated patients, were found to be resistant to isoniazid, rifampin, and streptomycin in combination.

**Table 4:** Distribution of multidrug resistance patterns, by history of tuberculosis treatment.

Drug combination	Multidrug resistance		History of tuberculosis treatment				OR (95% CI)	p
			No previous treatment		Previous treatment			
			(n = 211)		(n = 430)			
	n	%	n	%	n	%		
R+S	3	1.0	1	0.9	2	1.0	0.26 (0.0.8-0.78)	0.011
H+S	18	6.0	12	11.0	6	3.1	1.14 (0.08-32.04)	1.000
H+R	58	19.3	19	17.4	39	20.3	1.21 (0.63-2.32)	0.647
H+R+S	139	46.2	50	45.9	89	46.4	1.02 (0.62-1.68)	0.968
H+R+E	19	6.3	7	6.4	12	6.3	0.97 (0.34-2.83)	0.851
H+R+S+E	64	21.3	20	18.3	44	22.9	1.32 (0.71-2.49)	0.432
Total	301	100.0	109	100.0	192	100.0		

H : isoniazid

R : rifampin

S : streptomycin

E : ethambutol

## Discussion

Most of the MDR-TB patients were male and in the 21-40 year age bracket (Table 1). The high occurrence of MDR-TB among males and working-age individuals is probably due to the high number of Mozambican males working in South African mines, which constitute a high-risk environment for TB and other infectious diseases. These males often return to their home country whenever they become ill, thereby increasing the risk of infection among their wives and close contacts. The fact that working-age individuals constitute the most commonly affected age group is due to the fact that many such individuals, in search of better pay and, consequently, better living conditions, work in the close quarters of the aforementioned mines. Our results are consistent with those reported in other studies.^(^ ^3^ ^,^ ^16^ ^-^ ^21^ ^)^

The proportion of MDR-TB samples was higher than was that of non-MDR-TB samples, the difference being statically significant (p < 0.001). Most of the MDR-TB samples were from previously treated patients, and the fact that there were 2 XDR-TB samples shows that there might be more cases of XDR-TB not diagnosed as such. Therefore, further efforts are needed in order to improve diagnosis and treatment.

The results of the present study show that M. tuberculosis monoresistance to isoniazid and rifampin was most common in samples from previously treated patients, as was M. tuberculosis resistance to isoniazid, rifampin, and streptomycin in combination. Although previous treatment can influence the onset of resistance to isoniazid, rifampin, and streptomycin in combination, we found no statistically significant difference between previously treated and untreated patients regarding resistance to this drug combination. This finding is consistent with those of similar studies conducted in Brazil, Portugal, and Turkey,^(^ ^21^ ^-^ ^24^ ^)^ as well as with those of studies conducted in Mozambique, South Africa, Tanzania, Iran, and New Delhi.^(^ ^6^ ^,^ ^13^ ^,^ ^25^ ^-^ ^27^ ^)^

Treatment failure, poor adherence to treatment, and spontaneous mutations in *M*. *tuberculosis* strains probably played a major role in the emergence of MDR-TB, which can progress to XDR-TB.^(^ ^4^ ^,^ ^5^ ^,^ ^14^ ^,^ ^15^ ^,^ ^28^ ^)^ This could explain why the number of MDR-TB cases was higher among previously treated patients and shows that further efforts are needed to ensure rapid diagnosis of MDR-TB/XDR-TB and access to treatment with second-line drugs in Mozambique. In order to manage drug-resistant forms of TB and prevent further cases of MDR-TB and XDR-TB, a comprehensive approach similar to that used in cases of drug-susceptible TB is needed to ensure rapid detection and appropriate treatment, as are public health measures to cure patients and prevent further transmission of the disease,^(^ ^12^ ^,^ ^13^ ^,^ ^29^ ^,^ ^30^ ^)^ given that the epidemic of drug-resistant TB has spread.^(^ ^13^ ^,^ ^15^ ^,^ ^28^ ^)^

The classification of drug resistance as primary or acquired is used as an indicator of the efficacy of national tuberculosis control programs and in the adjustment and development of such programs. Unsupervised treatment can lead to an increase in the number of MDR-TB cases among previously treated patients. Although the directly observed treatment, short-course strategy has been adopted in Mozambique, the efficacy of this strategy needs to be evaluated. Cases of MDR-TB and XDR-TB must be effectively managed, second-line drugs being carefully used in order to reduce the morbidity, mortality, and transmission of MDR-TB and prevent the development of XDR-TB.^(^ ^12^ ^,^ ^15^ ^,^ ^29^ ^)^ In addition, better integration of the National Tuberculosis Control Program activities and activities such as counseling and home-based care could assist in controlling TB in the country.

In conclusion, antituberculosis drug resistance is high in laboratory-confirmed cases of TB in Mozambique, especially among previously treated patients. It is possible that XDR-TB strains are circulating in the population, given that we identified 2 XDR-TB cases in the present study (1 being in a previously treated patient and 1 being in a previously untreated patient). Resistance to isoniazid, rifampin, and streptomycin in combination was found to be high, particularly in previously treated patients.
