# Supplementary material for: Mycobacterium tuberculosis resistance to antituberculosis drugs in Mozambique
Source: J Bras Pneumol. 2014 Mar-Apr;40(2):142–7. doi: 10.1590/S1806-37132014000200007 (PMC4083649; doi:10.1590/S1806-37132014000200007)
Supplement: Translation

# Translation: Resistência de *Mycobacterium tuberculosis* aos tuberculostáticos em Moçambique*

**Authors:** Germano Manuel Pires, Elena Folgosa, Ndlovu Nquobile, Sheba Gitta, Nureisha Cadir

**Affiliations:** Instituto Nacional de Saúde, Ministério da Saúde de Moçambique, Maputo, Moçambique; Faculdade de Medicina da Universidade Eduardo Mondlane, Maputo, Moçambique; African Field Epidemiology Network, Kampala, Uganda; African Field Epidemiology Network, Kampala, Uganda; Laboratório Nacional de Referência da Tuberculose, Maputo, Moçambique

**Keywords:** Tuberculose extensivamente resistente a drogas, Tuberculose, Tuberculose resistente a múltiplos medicamentos

## Abstract

**OBJETIVO:**

Avaliar o perfil de resistência de *Mycobacterium tuberculosis* aos tuberculostáticos em Moçambique.

**MÉTODOS:**

Foram analisados dados secundários do Laboratório Nacional de Referência da Tuberculose, em Maputo, Moçambique, e do Laboratório Regional de Referência da Tuberculose, na Beira, Moçambique. Os dados foram relativos a amostras positivas à cultura e submetidas ao teste de sensibilidade aos tuberculostáticos de primeira linha durante o período de janeiro a dezembro de 2011. Os resultados do teste de sensibilidade foram analisados, e sua frequência foi comparada com o tipo de paciente e sua proveniência.

**RESULTADOS:**

Foram analisadas 641 cepas, isoladas em cultura e submetidas ao teste de sensibilidade. Das 641 cepas, 374 (58,3%) foram resistentes a pelo menos um tuberculostático e 280 (43,7%) revelaram-se multirresistentes. Dos 280 casos de tuberculose multirresistente, 184 (65,7%) eram pacientes com tratamento prévio, a maioria dos quais era oriunda da zona sul do país. Confirmou-se que 2 (0,71%) dos casos de tuberculose multirresistente eram casos de tuberculose extensivamente resistente a drogas. O sexo masculino foi o mais afetado, particularmente na faixa etária de 21 a 40 anos.

**CONCLUSÕES:**

A resistência de *M*. *tuberculosis* aos tuberculostáticos é elevada em Moçambique, especialmente em indivíduos com tratamento prévio. A resistência de *M*. *tuberculosis* à combinação de isoniazida, rifampicina e estreptomicina foi elevada, especialmente em amostras provenientes de indivíduos com tratamento prévio.

## Introdução

A tuberculose (TB) ainda é um sério problema de saúde pública em muitos países de baixa e média renda na África, na Ásia e na antiga União Soviética.^(^ ^1^ ^)^ De acordo com a Organização Mundial da Saúde, são registrados cerca de 9 milhões de novos casos de TB a cada ano no mundo, dos quais 4 milhões são casos de TB infecciosa. Em muitos países, o número de casos de TB quadruplicou, não obstante a implantação de estratégias eficazes para combater a doença.^(^ ^2^ ^)^

Aumentou não só o número de estudos sobre a resistência aos tuberculostáticos (de 1 em 2008 para 10 em 2011) como também o número de países que forneceram dados representativos sobre a resistência a medicamentos (de 19 para 22), Moçambique inclusive.^(^ ^3^ ^)^ A falha de tratamento, a baixa adesão ao tratamento e mutações espontâneas em cepas de *Mycobacterium tuberculosis* têm contribuído para o surgimento de novos casos de TB multirresistente, que pode posteriormente evoluir para TB extensivamente resistente.^(^ ^4^ ^-^ ^6^ ^)^ Estudos recentes sobre a resistência a medicamentos identificaram altos índices de TB multirresistente no sul da África, e, até o final de 2010, 69 países (Moçambique inclusive) relataram pelo menos um caso de TB extensivamente resistente.^(^ ^2^ ^,^ ^7^ ^,^ ^8^ ^)^ Em Moçambique, a TB resistente a medicamentos é um grande problema. A pesquisa nacional sobre a resistência aos tuberculostáticos em Moçambique em 2008 mostrou que, de todos os casos de TB multirresistente, 3,5% eram casos de TB recentemente diagnosticada e 11,2% ocorreram em indivíduos que já haviam recebido tratamento antituberculose; em 2011, foram detectados 47.452 casos de todas as formas de TB,^(^ ^3^ ^)^ embora nenhum deles fosse de TB extensivamente resistente.

Em virtude do crescente número de casos de TB multirresistente^(^ ^4^ ^)^ e do surgimento da TB extensivamente resistente em Moçambique, buscamos avaliar a resistência de *M*. *tuberculosis* aos tuberculostáticos em pacientes com TB previamente tratados e não tratados em Moçambique. Buscamos também determinar a magnitude da resistência aos tuberculostáticos no país, a fim de informar o Programa Nacional de Controle da Tuberculose a respeito da eficácia das medidas de controle da TB e do tratamento antituberculose e elaborar esquemas e estratégias eficazes de tratamento para todos os pacientes com TB no país.

## Métodos

Trata-se de um estudo transversal baseado em dados laboratoriais secundários relativos ao período de janeiro a dezembro de 2011. Os dados foram relativos a 641 culturas positivas para TB provenientes do Laboratório Nacional de Referência da Tuberculose, na cidade de Maputo, em Moçambique, e do Laboratório Regional de Referência da Tuberculose em Beira, Moçambique. Esses dois laboratórios servem a todas as 11 províncias de Moçambique.

Foram considerados casos de TB multirresistente todos os indivíduos infectados por um isolado resistente a pelo menos isoniazida e rifampicina; casos confirmados de TB extensivamente resistente foram excluídos.^(^ ^9^ ^-^ ^11^ ^)^ Foram considerados casos de TB extensivamente resistente os casos de TB multirresistente resistentes a uma fluoroquinolona e a um medicamento injetável de segunda linha que não a estreptomicina.^(^ ^12^ ^-^ ^15^ ^)^ Pacientes com TB multirresistente ou extensivamente resistente registrados para receber tratamento sem nunca ter recebido tratamento antituberculose durante mais de 4 semanas foram considerados casos de resistência primária. Pacientes com TB multirresistente/extensivamente resistente em retratamento cujo primeiro episódio de TB ocorrera antes de 2011 foram considerados casos de resistência adquirida.

Todas as amostras foram submetidas a baciloscopia e cultura. Todas as amostras positivas à cultura foram submetidas ao teste de sensibilidade aos medicamentos de primeira linha, realizado por meio do método da razão. Das amostras de TB multirresistente, 71 foram enviadas ao Laboratório Supranacional de Referência da TB em Milão, na Itália, para o teste de sensibilidade aos medicamentos de segunda linha.

Os dados secundários do Laboratório Nacional de Referência da Tuberculose (dados demográficos e resultados do teste de sensibilidade) provieram de um banco de dados WixDisa, versão 04.16.04.652 (Disa, África do Sul), e do livro de registros do laboratório, ao passo que os do Laboratório Regional de Referência da Tuberculose em Beira provieram de um banco de dados Microsoft Excel. Todos os resultados positivos de cultura e do teste de sensibilidade foram inseridos em um banco de dados Epi Info 3.5.1 e analisados. Calculamos as OR e seus IC95% para determinar a relação de pacientes previamente tratados e não tratados com os resultados do teste de sensibilidade.

O estudo foi analisado e aprovado pelo Comitê de Ética em Pesquisa do Instituto Nacional de Saúde, em Maputo, Moçambique. Não foi necessária a assinatura de um termo de consentimento livre e esclarecido, pois o estudo baseou-se em dados secundários e não tivemos acesso a nenhuma informação que permitisse a identificação dos pacientes.

## Resultados

Durante o período de estudo, foram analisadas 641 amostras positivas à cultura para TB (561 amostras do Laboratório Nacional de Referência da Tuberculose e 80 do Laboratório Regional de Referência da Tuberculose em Beira). A Tabela 1 mostra a distribuição dos pacientes com TB multirresistente e extensivamente resistente de acordo com o gênero e a faixa etária.

**Tabela 1:** Distribuição de pacientes com tuberculose multirresistente e extensivamente resistente de acordo com o gênero e a faixa etária.

Faixa etária (anos)	Pacientes com TB multirresistente			Pacientes com TB extensivamente resistente		
	(n = 280)			(n = 2)		
	Gênero		Total	Gênero		Total
	F	M		F	M	
	(n)	(n)		(n)	(n)	
0-20	16	3	19			
21-40	92	99	191	1	1	2
41-60	20	36	56			
> 60	1	4	5			
Dados incompletos	3	6	9			
Total	132	148	280	1	1	2

TB : tuberculose

F : feminino

M : masculin

Das 641 amostras, 430 (67,1%) provieram de pacientes com TB previamente tratados e 280 (43,7%) provieram de pacientes com TB multirresistente (Tabela 2). Dessas 280 amostras, 148 (53%) provieram de homens e 191 (68,2%) provieram de indivíduos na faixa etária de 21-40 anos. Houve 2 pacientes com TB extensivamente resistente, ambos na faixa etária de 21-40 anos.

**Tabela 2:** Distribuição de pacientes com tuberculose multirresistente e não multirresistente de acordo com a história de tratamento antituberculose.

História de tratamento	Pacientes com TB multirresistente	Pacientes com TB não multirresistente	OR	p
	n (%)	n (%)		
Tratamento prévio	184 (65,7)	172 (47,6)	2,06 (1,47-2,88)	< 0,001
Sem tratamento prévio	96 (34,3)	185 (51,2)		
Dados incompletos		4 (1,1)		
Total	280 (100)	361 (100)		

TB : tuberculose.

Confirmou-se que, das amostras de TB multirresistente enviadas ao Laboratório Supranacional de Referência da TB para a realização do teste de sensibilidade aos medicamentos de segunda linha, 2 eram de TB extensivamente resistente, das quais 1 provinha de um paciente que já havia recebido tratamento e 1 provinha de um paciente que nunca havia recebido tratamento (Figura 1).

**Figura 1:** Proporção de casos com tuberculose multirresistente (MDR) e extensivamente resistente (XDR) de acordo com a história de tratamento antituberculose.

Dentre as 641 amostras avaliadas, o padrão mais comum de resistência a medicamentos foi monorresistência a isoniazida (em 5,8%), seguido de monorresistência a rifampicina (3,4%). A Tabela 3 mostra a distribuição dos padrões de resistência a medicamentos específicos de acordo com a história de tratamento antituberculose.

**Tabela 3:** Distribuição dos padrões de resistência a medicamentos específicos de acordo com a história de tratamento antituberculose.

Monorresistência			História de tratamento				OR (IC95%)	p
			Sem tratamento prévio		Tratamento prévio			
			(n = 211)		(n = 430)			
Droga	n	%	n	%	n	%		
H	37	56,9	17	53,1	20	60,6	1,36 (0,45-4,09)	0,720
R	22	33,8	10	31,3	12	36,4	1,26 (0,40-4,00)	0,862
S	2	3,1	1	3,1	1	3,0	0,97 (0,03-37,42)	1,000
E	4	6,2	4	12,5	0	0,0	-	0,053
Total	65	100,0	32	100,0	33	100,0		

H : isoniazida

R : rifampicina

S : estreptomicina

E : etambutol

A Tabela 4 mostra a distribuição dos padrões de resistência a múltiplos medicamentos de acordo com a história de tratamento antituberculose. A maioria das amostras de *M*. *tuberculosis*, particularmente aquelas provenientes de pacientes previamente tratados, apresentou resistência à combinação de isoniazida, rifampicina e estreptomicina.

**Tabela 4:** Distribuição dos padrões de resistência a múltiplos medicamentos de acordo com a história de tratamento antituberculose.

Associação medicamentosa	Multirresistência		História de tratamento				OR (IC95%)	p
			Sem tratamento prévio		Tratamento prévio			
			(n = 211)		(n = 430)			
	n	%	n	%	n	%		
R+S	3	1,0	1	0,9	2	1,0	0,26 (0,08-0,78)	0,011
H+S	18	6,0	12	11,0	6	3,1	1,14 (0,08-32,04)	1,000
H+R	58	19,3	19	17,4	39	20,3	1,21 (0,63-2,32)	0,647
H+R+S	139	46,2	50	45,9	89	46,4	1,02 (0,62-1,68)	0,968
H+R+E	19	6,3	7	6,4	12	6,3	0,97 (0,34-2,83)	0,851
H+R+S+E	64	21,3	20	18,3	44	22,9	1,32 (0,71-2,49)	0,432
Total	301	100,0	109	100,0	192	100,0		

H : isoniazida

R : rifampicina

S : estreptomicina

E : etambutol

## Discussão

A maioria dos pacientes com TB multirresistente era do sexo masculino e estava na faixa etária de 21-40 anos (Tabela 1). A elevada ocorrência de TB multirresistente em homens e indivíduos em idade produtiva provavelmente se deve ao elevado número de homens moçambicanos que trabalham nas minas sul-africanas, as quais constituem um ambiente de alto risco de TB e outras doenças infecciosas. Esses homens muitas vezes retornam a seu país de origem sempre que adoecem, aumentando assim o risco de infecção em suas esposas e contatos próximos. O fato de que a faixa etária mais afetada é a dos indivíduos em idade produtiva deve-se ao fato de que muitos desses indivíduos, em busca de melhores salários e, consequentemente, melhores condições de vida, trabalham confinados nas minas supracitadas. Nossos resultados vão ao encontro daqueles relatados em outros estudos.^(^ ^3^ ^,^ ^16^ ^-^ ^21^ ^)^

A proporção de amostras de TB multirresistente foi maior que a de amostras de TB não multirresistente, e a diferença foi estatisticamente significante (p < 0,001). A maioria das amostras de TB multirresistente proveio de pacientes previamente tratados, e as 2 amostras de TB extensivamente resistente mostram que pode haver mais casos de TB extensivamente resistente não diagnosticados como tal. Portanto, são necessários mais esforços para melhorar o diagnóstico e o tratamento.

Os resultados do presente estudo mostram que a monorresistência de* M*. *tuberculosis* a isoniazida e rifampicina foi mais comum em amostras provenientes de pacientes previamente tratados, assim como o foi a resistência de *M*. *tuberculosis* à combinação de isoniazida, rifampicina e estreptomicina. Embora o tratamento prévio possa influenciar o aparecimento de resistência à combinação de isoniazida, rifampicina e estreptomicina, não observamos diferença estatisticamente significante entre pacientes previamente tratados e não tratados no que tange à resistência a essa associação medicamentosa. Esse resultado vai ao encontro dos de estudos semelhantes realizados no Brasil, em Portugal e na Turquia,^(^ ^21^ ^-^ ^24^ ^)^ bem como dos de estudos realizados em Moçambique, na África do Sul, na Tanzânia, no Irã e em Nova Déli.^(^ ^6^ ^,^ ^13^ ^,^ ^25^ ^-^ ^27^ ^)^

A falha de tratamento, a baixa adesão ao tratamento e mutações espontâneas em cepas de *M*. *tuberculosis* provavelmente desempenharam um papel importante no surgimento da TB multirresistente, que pode evoluir para TB extensivamente resistente.^(^ ^4^ ^,^ ^5^ ^,^ ^14^ ^,^ ^15^ ^,^ ^28^ ^)^ Isso poderia explicar por que o número de casos de TB multirresistente foi maior entre os pacientes previamente tratados e mostra que são necessários mais esforços para assegurar o diagnóstico rápido de TB multirresistente ou extensivamente resistente e o acesso ao tratamento com medicamentos de segunda linha em Moçambique. Para lidar com as formas resistentes da TB e prevenir novos casos de TB multirresistente e extensivamente resistente, é necessária uma abordagem abrangente semelhante àquela empregada em casos de TB sensível a medicamentos para garantir rápida detecção e tratamento adequado, assim como o são medidas de saúde pública para curar os pacientes e prevenir maior transmissão da doença,^(^ ^12^ ^,^ ^13^ ^,^ ^29^ ^,^ ^30^ ^)^ já que a epidemia de TB resistente a medicamentos se espalhou.^(^ ^13^ ^,^ ^15^ ^,^ ^28^ ^)^

A classificação da resistência a medicamentos em primária ou adquirida é usada como um indicador da eficácia dos programas nacionais de controle da tuberculose e na elaboração e adaptação de tais programas. O tratamento não supervisionado pode levar a um aumento dos casos de TB multirresistente em pacientes previamente tratados. Embora a estratégia de tratamento diretamente observado tenha sido adotada em Moçambique, sua eficácia precisa ser avaliada. Os casos de TB multirresistente e extensivamente resistente devem ser efetivamente tratados, sendo os medicamentos de segunda linha usados com cautela a fim de reduzir a morbidade, mortalidade e transmissão da TB multirresistente e prevenir o surgimento da TB extensivamente resistente.^(^ ^12^ ^,^ ^15^ ^,^ ^29^ ^)^ Além disso, uma maior integração das atividades do Programa Nacional de Controle da Tuberculose a atividades como o aconselhamento e o cuidado domiciliar poderia ajudar a controlar a TB no país.

Em suma, a resistência aos tuberculostáticos é elevada em casos de TB confirmados laboratorialmente em Moçambique, especialmente em pacientes previamente tratados. É possível que cepas de TB extensivamente resistente estejam circulando na população, já que foram identificados 2 casos de TB extensivamente resistente no presente estudo (1 em um paciente previamente tratado e 1 em um paciente sem tratamento prévio). A resistência à combinação de isoniazida, rifampicina e estreptomicina foi elevada, particularmente em pacientes previamente tratados.
